# An online intervention designed to reduce self‐stigma and increase help‐seeking in Arabic‐speaking refugees with posttraumatic stress symptoms: A randomized controlled trial

**DOI:** 10.1002/jts.23168

**Published:** 2025-05-30

**Authors:** Natalie Mastrogiovanni, Angela Nickerson

**Affiliations:** ^1^ School of Psychology, University of New South Wales Sydney Australia

## Abstract

Despite elevated rates of psychopathology, refugees underutilize mental health services. Mental health self‐stigma is a prominent barrier to accessing psychological support; however, there is limited research on intervention approaches to reduce self‐stigma among refugees. The present study aimed to provide further support for the Tell Your Story (TYS) intervention in reducing self‐stigma and increasing help‐seeking among Arabic‐speaking male and female refugees. In this randomized controlled trial (RCT), 67 Arabic‐speaking refugees with self‐stigma and at least subthreshold posttraumatic stress symptoms (PTSS) were randomly allocated to the TYS group or waitlist control group. At baseline, postintervention, and 3‐month follow‐up, participants completed assessment measures indexing measures of self‐stigma (related to symptoms and help‐seeking) and help‐seeking (intentions and behavior). Poisson regression analyses revealed that participants in the TYS group demonstrated more help‐seeking behavior at 3‐month follow‐up than those in the waitlist control group, Hedges’ *g* = 0.67. However, linear mixed models showed that the waitlist control group demonstrated larger decreases in PTSD‐related self‐stigma across time, T2: *g* = 0.07, T3: *g* = 0.04, whereas no significant group differences were observed for self‐stigma related to help‐seeking. Although the findings were mixed and suggest a need for further investigation in a larger RCT with a sample of refugee men and women, the results provide support for the intervention's utility in expanding one's help‐seeking network in a population with low treatment uptake.

There are currently over 117,000,000 forcibly displaced people worldwide who have been forced to flee their home country due to war, violence, conflict, and/or persecution (United Nations High Commissioner for Refugees [UNHCR], 2024). As such, refugees experience numerous traumatic events in their home country and through the process of fleeing to seek asylum. Cumulative trauma exposure and exposure to interpersonal trauma (e.g., torture, sexual assault), in particular, confer an increased risk of psychopathology, especially posttraumatic stress disorder (PTSD), among refugees (Neuner et al., 2004; Patanè et al., 2022; Steel et al., 2009). These elevated rates of psychopathology underscore the importance of host countries supporting refugees in their recovery. However, the rates of seeking help from mental health professionals (i.e., formal help‐seeking) are relatively low despite the availability of evidence‐based treatments and services for refugees. For example, in Australia, the Forum of Australian Services for Survivors of Torture and Trauma (FAASTT) network consists of specialist services for survivors of torture and trauma. Despite this, refugees significantly underutilize mental health services in comparison to citizens of the host country (Mazumdar et al., 2022; Zheng et al., 2022). Additionally, feelings of shame, fear of burdening others, and/or difficulty trusting others may reduce help‐seeking from informal supports, such as friends and family (i.e., informal help‐seeking; de Anstiss & Ziaian, 2010; Slobodin et al., 2018). Accordingly, the disparity between mental health needs and help‐seeking warrants further attention.

Research consistently demonstrates that self‐stigma is a key barrier to refugees accessing support (Byrow et al., 2019). Self‐stigma occurs when stigmatizing views in relation to symptoms or seeking help are internalized and applied to the self (Bär et al., 2021; Gaebel et al., 2017). Studies have demonstrated an association between increased self‐stigma and reduced help‐seeking in both the general population and refugees (Byrow et al., 2019; Clement et al., 2015; Özaslan et al., 2024). For example, Byrow and colleagues (2019) found that self‐stigma of PTSD (SS‐PTSD) underlies the association between PTSD and reduced informal help‐seeking, whereas self‐stigma of help‐seeking (SS‐HS) underlies the association between PTSD and reduced formal and informal help‐seeking among refugee men. Similar findings were observed in a sample of refugee youth, revealing that SS‐HS fully mediated the association between mental health difficulties and help‐seeking intentions (Özaslan et al., 2024).

Reducing self‐stigma is, therefore, a critical intervention target to increase treatment uptake. To our knowledge, only one intervention has been designed specifically to reduce self‐stigma and increase help‐seeking among refugees. This online intervention, “Tell Your Story” (TYS; Nickerson et al., 2020), implements evidence‐based stigma reduction strategies, including psychoeducation, cognitive reappraisal, and social contact (Alonso et al., 2019; Yanos et al., 2015) to reduce self‐stigma related to PTSD. The online delivery method reduces engagement barriers associated with stigma by affording users anonymity (Lindegaard et al., 2021). In a randomized controlled trial (RCT), TYS was associated with smaller increases in SS‐HS as well as increased help‐seeking behavior at 1‐month follow‐up compared to a waitlist control condition in a sample of 103 refugee men (Nickerson et al., 2020).

Though these findings suggest that the intervention represents a promising approach to reducing self‐stigma among refugee men, two outstanding questions remain following this study. First, it is unclear as to whether the intervention would also be effective when women are included in the sample. Although PTSD prevalence is higher among refugee women compared to men, which increases the risk of self‐stigma (Blackmore et al., 2020; Kira et al., 2014), male refugees are overrepresented in samples investigating internet‐based interventions (El‐Haj‐Mohamad et al., 2023). Thus, the inclusion of women in study samples is necessary to determine whether internet‐based modalities are appropriate across genders. Second, it remains to be seen whether changes in stigma and help‐seeking are maintained beyond the brief 1‐month follow‐up period in the previous study. Given the mixed evidence for the effectiveness of mental health stigma interventions beyond a 1‐month follow‐up period among people with mental health difficulties (Thornicroft et al., 2016), there is a need to determine the longevity of changes.

Using an RCT design, the aim of the current study was to replicate and extend the previous findings on the effectiveness of TYS (Nickerson et al., 2020). Specifically, we aimed to investigate whether the TYS intervention would reduce self‐stigma and increase help‐seeking in a sample of male and female refugee participants with posttraumatic stress symptoms (PTSS). We hypothesized that participants in the TYS group would demonstrate larger decreases in self‐stigma (i.e., both SS‐PTSD and SS‐HS) and larger increases in help‐seeking intentions and behavior relative to those assigned to a waitlist control (WLC) condition across time.

## METHOD

### Participants

Participants were 67 male and female refugees (TYS: *n =* 33, WLC: *n* = 34) resettled in Australia. The inclusion criteria were having a refugee or asylum‐seeker background, being literate (i.e., able to read and write) in Arabic, having internet access, and being over 18 years of age. Participants were also required to have at least subthreshold levels of probable PTSD, as assessed using the Primary Care PTSD Screen for *DSM‐5* (PC‐PTSD‐5; Prins et al., 2016), and to endorse self‐stigma related to mental health or help‐seeking, defined as a response of “neutral,” “agree,” or “strongly agree” on at least one of four selected items from the Self‐Stigma of Seeking Help Scale (Vogel et al., 2006) and the Internalized Stigma of Mental Illness Scale (Ritsher et al., 2003).

Potential participants who endorsed active suicidality (i.e., reporting a suicidal plan or intent) were excluded from the study. Recruitment for this study took place between May 2022 and July 2023. Participants were recruited from several sources, including advertising at refugee services, social media platforms, and snowball sampling wherein participants were asked to indicate if someone they knew would be interested in participating. Additionally, participants were recruited from English classes in Australia delivered as part of the Adult Migrant English Program, where information about the study was verbally presented to each class, and participants who indicated interest in participating were contacted.

### Procedure

#### Recruitment and screening

Interested participants completed the eligibility screener either online or via telephone, depending on participant preference, with a psychologist. The verbal format circumvented any technological difficulties, whereas the online format was an option for participants who may have presented with pronounced stigma and, therefore, were reluctant to discuss psychological symptoms with others. If participants were eligible based on the screening survey, they received a telephone call from a psychologist to screen for active suicidality and confirm interest in participating.

#### Randomization and assessments

Eligible participants were emailed a link to the baseline survey and required to provide consent electronically for the whole study before completing the baseline survey. After completing the baseline survey, participants were randomized either to the TYS group or the WLC group. Randomization was based on a 1:1 allocation ratio, with two groups, and conducted using a computerized program. To ensure balance between groups, the allocation was stratified by gender (male vs. female). Participants in the TYS condition had immediate access to the intervention for 4 weeks. Because the intervention is self‐paced yet time‐limited, reminders to complete the intervention were sent via email if participants were not completing enough chapters each week to encourage completion within the 4‐week access period. Individuals in the WLC group were notified that they could access the intervention upon completion of their final survey (i.e., 3‐month follow‐up assessment). The postintervention survey was sent via email 4 weeks after baseline, and the 3‐month follow‐up assessment was sent via email 3 months after the postintervention (i.e., 4 months after baseline). The baseline assessment included a battery of questionnaires along with a demographic survey, and the subsequent assessments included the battery of questionnaires except for the demographic survey and the measure of trauma exposure. If participants had not completed an assessment after 1 week, they were reminded to complete the survey once per week for 5 weeks. Participants were provided with a $25 (AUD) voucher upon the completion of each assessment. The trial was prospectively registered on the Australia and New Zealand Clinical Trial Registry (Trial ID ACTRN12621001731886). As specified on the trial registry, primary outcomes included SS‐HS, SS‐PTSD, and help‐seeking intentions and behavior.

All measures were translated into Arabic and blind‐back translated by accredited translators experienced in working with mental health–related material. The translators and research team reconciled any minor discrepancies. As the intervention focuses on reducing mental health stigma related to PTSS specifically, we adapted outcome measures to be relevant for PTSS when possible (as noted in the Measures section). However, for outcome measures that focused on help‐seeking, we maintained a broad focus rather than restricting items solely to PTSS. For example, for the Self‐Stigma of Seeking Help Scale, we retained the item, “My self‐confidence would be threatened if I sought professional help” rather than amending the item to read “My self‐confidence would be threatened if I sought professional help for PTSD.” The reason for this was two‐fold. First, psychological treatment often addresses comorbid mental health conditions rather than focusing on a single type of presentation, thus anchoring the self‐stigma of psychological treatment to PTSD is unlikely to accurately reflect the focus of treatment in the real world. Second, and relatedly, clients may be likely to seek help based on a constellation of symptoms (e.g., sleep difficulties, nightmares, low mood) without specifically recognizing that these are representative of a particular disorder. Accordingly, amending help‐seeking scales to specifically focus on PTSD may lead to the underreporting of help‐seeking intentions and behaviors.

#### TYS intervention

The TYS intervention (Nickerson et al., 2020) consisted of information, short videos, and interactive activities organized into 11 short modules. Each module took approximately 10–15 min to complete. Modules either had a topic that targeted self‐stigma or help‐seeking and implemented evidence‐based stigma reduction strategies, including social contact, psychoeducation, cognitive reappraisal of PTSD symptoms, and help‐seeking concerns. Participants progressed through the intervention in a self‐paced manner, completing modules chronologically. See Supplementary Table S1 for further information and an explanation of how the intervention was adapted to be applicable to female participants.

### Measures

#### PTSS screener

As part of the eligibility screening survey, participants completed the PC‐PTSD‐5 (Prins et al., 2016). The PC‐PTSD‐5 included a single question indexing trauma exposure, followed by five items representing the four PTSD symptom clusters outlined in the *Diagnostic and Statistical Manual of Mental Disorders* (5th ed.; *DSM‐5*; American Psychiatric Association, 2013): intrusions, avoidance, negative alterations in cognition and mood, and hyperarousal. Participants responded “yes” or “no” to all items and were considered to meet the criteria for subthreshold PTSD if they endorsed the Cluster B (intrusions) item along with at least one other item in the screener.

#### Exposure to potentially traumatic events

Exposure to potentially traumatic events (PTEs) was assessed using an adapted 20‐item version of the Harvard Trauma Questionnaire (HTQ; Mollica et al., 1992) that indexed different types of PTEs commonly experienced by refugees (e.g., imprisonment, combat situation, lack of food or water, physical assault). The measure is widely used among refugees and has demonstrated good reliability and validity (Sigvardsdotter et al., 2016). Participants indicated whether they had either experienced, witnessed, or learned about each event, with responses of “experienced” or “witnessed” considered PTE endorsement. Affirmative responses were summed to create a count indexing PTE diversity exposure (range: 0–20).

#### PTSS

Posttraumatic stress symptoms were measured using the 20‐item Posttraumatic Diagnostic Scale–5 (PDS; Foa et al., 2016). The PDS has been validated in Arabic, used in refugee samples, and shown to demonstrate strong reliability and validity (Alghamdi & Hunt, 2019; Selmo et al., 2019). Items reflect PTSD symptoms included in the four *DSM‐5* symptom clusters. Participants indicated how much each symptom bothered them over the past month, rating responses on a 5‐point scale ranging from 0 (*not at all*) to 4 (*6 or more times a week/severe*). An item‐level mean score was created. In this sample, internal consistency for the scale in this study was excellent, Cronbach's α = .94.

#### SS‐PTSD

An adapted version of the Internalized Stigma of Mental Illness Scale (Ritsher et al., 2003) was used to assess self‐stigma related to PTSD. The scale consisted of 24 items capturing self‐stigmatizing beliefs about PTSD symptoms (e.g., “I am embarrassed or ashamed that I have symptoms of posttraumatic stress”). A total self‐stigma of PTSD score was calculated along with scores on four subscales assessing different dimensions of self‐stigma: Alienation, Stereotype Endorsement, Discrimination Experience, and Social Withdrawal. The original measure includes a Stigma Resistance subscale, which was excluded from the current study due to poor internal consistency with the broader scale in other studies; however, the 24‐item version has still demonstrated strong reliability (Ritsher et al., 2003; Wastler et al., 2020). Participants were asked to indicate the extent to which they endorsed each belief on a 4‐point scale ranging from 1 (*strongly disagree*) to 4 (*strongly agree*). The item‐level mean was used in this study, with higher scores reflecting higher levels of SS‐PTSD. In this sample, internal consistency for the scale was excellent, Cronbach's α = .95.

#### SS‐HS

An adapted version of the Self‐Stigma of Seeking Help Scale (Vogel et al., 2006) was used to measure SS‐HS. The scale has demonstrated strong psychometric properties, namely reliability and validity, and has previously been used in studies with Arabic‐speaking refugees (Byrow et al., 2019; Mastrogiovanni et al., 2024; Vogel et al., 2006). The scale consisted of 10 items assessing self‐stigmatizing beliefs toward seeing a professional for mental health difficulties. In the original version, five items were measured in the negative direction (e.g., “If I went to a therapist I would be less satisfied with myself”), and five items were measured in the opposite direction (e.g., “My self‐confidence would not be threatened if I sought professional help”). Given that previous cross‐cultural research has demonstrated that reverse‐scored items demonstrate poor internal consistency (Mastrogiovanni et al., 2024; Nickerson et al., 2015; Schlechter et al., 2021), items were all worded in the negative direction (e.g., “My self‐confidence would be threatened if I sought professional help”). Participants indicated the extent to which they agreed with each belief on a 5‐point scale ranging from 1 (*strongly disagree*) to 5 (*strongly agree*). The item‐level mean was calculated, whereby higher scores indicated higher levels of SS‐HS. Internal consistency for this scale was very good, Cronbach's α = .92.

#### Help‐seeking intentions

Intentions to seek help were measured using an adapted version of the General Help‐Seeking Questionnaire (GHSQ; Wilson et al., 2005), which has demonstrated good reliability and validity. Items measured how likely participants were to access help over the next 4 weeks from 12 potential help‐seeking sources, organized into three categories: a mental health professional (e.g., psychologist, general practitioner), another professional (e.g., caseworker, teacher), or an informal source (e.g., friend, spouse, partner, son/daughter, other family member, community leader, religious leader). Participants also had the option to select “other.” Help‐seeking sources from the original measure were adapted to better reflect potential sources of help available to refugees within the Australian context (e.g., “social worker” was replaced with “caseworker”), consistent with other refugee studies that have used this scale with strong reported reliability (Byrow et al., 2019; Nickerson et al., 2020). Participants responded on a 7‐point scale ranging from 1 (*extremely unlikely*) to 7 (*extremely likely*). The item‐level mean was calculated, with higher scores reflecting higher degrees of intention to seek help. In this sample, internal consistency for the scale was good, Cronbach's α = .87.

#### Help‐seeking behavior

Help‐seeking behavior was measured using an adapted version of the Actual Help‐Seeking Questionnaire (AHSQ; Rickwood et al., 2005), which has demonstrated good reliability and validity in previous studies. Participants indicated (i.e., “yes” or “no”) whether they had sought help from the same sources as listed on the GHSQ over the last 4 weeks at the baseline and postintervention assessments and over the last 3 months at follow‐up to capture periods between measurement points. Additionally, participants had the option to indicate “other” for each category (i.e., formal, other professional, and informal sources). For this study, total scores of the number of new help‐seeking sources accessed from baseline to postintervention and from baseline to follow‐up were calculated. This was achieved by using a difference score, which was calculated for each type of help‐seeking source accessed at postintervention compared to baseline and at follow‐up compared to baseline, which excluded sources of help previously sought at baseline. The difference score was summed yielding a total score of the number of new help‐seeking sources (range: 0–14).

#### Program usability

Program usability was assessed using 13 items indexing various aspects of the intervention's format based on the measure used in the original RCT (Nickerson et al., 2020). For information on program usability, please see the Supplementary Materials.

### Data analysis

A series of analyses was conducted to determine the impact of TYS on outcomes. The previous RCT informed power calculations using the PowerUp tool (Dong & Maynard, 2013). A total sample size of 100 participants was required to detect a between‐group effect size of 0.40 for self‐stigma at 80% power with an alpha value of .50, consistent with the findings reported by Nickerson and colleagues (2020).

First, independent samples *t* tests and chi‐square tests were performed to determine if there were any baseline differences in outcome variables and demographic characteristics. Next, a series of linear mixed models were analyzed in SPSS (Version 29) to determine if patterns of change across time for continuous outcome variables (SS‐PTSD, SS‐HS, help‐seeking intentions) differed between groups. In each linear mixed model, time was specified as a Level 1 predictor, where baseline was coded as 1, postintervention was coded as 2, and follow‐up was coded as 5 to represent the gap (in months) from the first time point to the last time point. Group was specified as a Level 2 predictor (0 = TYS, 1 = WLC, with the latter representing the reference group). Interaction terms were included in the model (Group x Time) to investigate whether patterns of change over time differed according to group assignment. Random intercepts were included in all models. Between‐group effect sizes (Hedges’ *g*) were calculated by using the mean difference between groups and dividing by the pooled standard deviation at each time point. Effect size values of 0.2 constituted a small effect size, 0.5 constituted a medium effect size, and 0.8 constituted a large effect size (Cohen, 2013). Missing data from baseline to follow‐up was 17.5%. There were no systematic differences in baseline variables between participants who did and did not complete the follow‐up assessment, leading us to conclude that the data were missing completely at random. We used restricted maximum likelihood estimation methods, which are appropriate for data missing completely at random and missing at random (Shin et al., 2017).

A different approach was required to analyze help‐seeking behavior given it was a count variable rather than a continuous variable. As such, Poisson regression analyses were conducted in SPSS (Version 29) to analyze change in help‐seeking behavior over time. Poisson regression analyses were conducted to investigate whether group assignment predicted new help‐seeking sources at postintervention and follow‐up (0 = TYS, 1 = WLC).

Consistent with intent‐to‐treat analyses, all participants who were randomized were included regardless of engagement with the intervention provided they completed the assessment for at least one time point. Given that approximately one third (*n* = 11) of the participants allocated to the TYS group did not start the intervention (i.e., noncompleters), we included a subsequent analysis that excluded these participants (see Supplementary Table S3).

## RESULTS

### Participant characteristics

**FIGURE 1:**
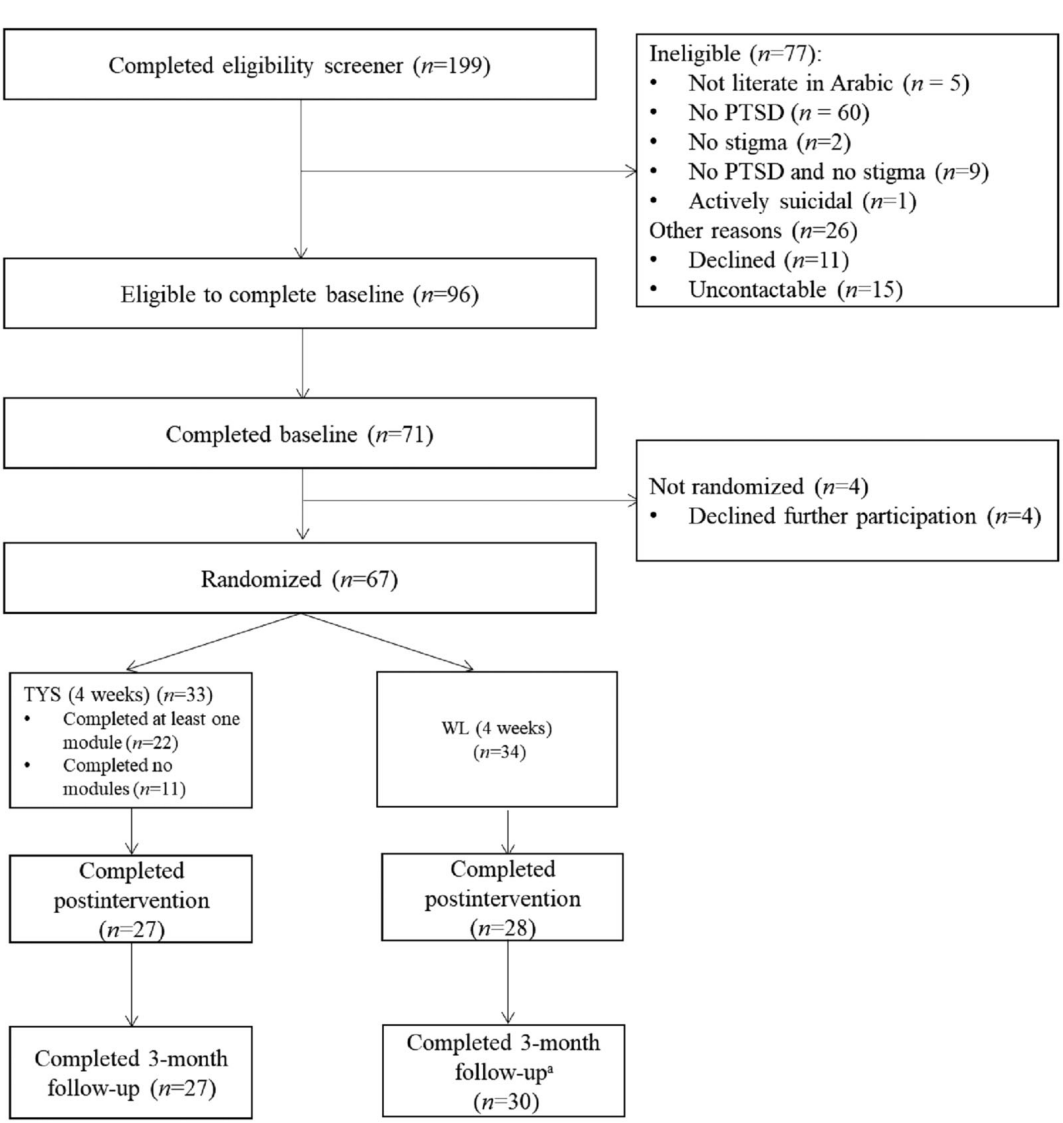
Participant flow throughout the study *Note*: Participant flow is represented in accordance with Consolidated Standards of Reporting Trials (CONSORT) guidelines (Schulz et al., 2010). ^a^Participants in the waitlist (WL) control group received access to the Tell Your Story (TYS) intervention after completing the 3‐month follow‐up assessment. Reasons for attrition at each assessment point (i.e., baseline, postintervention, and 3‐month follow‐up) included technological difficulties, other commitments, medical reasons and being uncontactable. PTSD = posttraumatic stress disorder.

A total of 67 participants were recruited. See Figure 1 for participant flow through the study, including losses and exclusions. The final number of participants recruited fell short of the initial target of 100 given challenges in recruitment. Briefly, participating in an intervention for a stigmatized problem (i.e., mental health) can be considered to be a form of help‐seeking, which can deter participation in research (Lindheimer et al., 2020). Limitations in funding also meant that we had to conclude recruitment at this point. Given the small final sample size, the present study is more consistent with a pilot‐level design. As such, the following results can only provide preliminary data on outcome measures. Additionally, factors affecting recruitment, as well as other issues related to the study, are further explored in the Discussion section with the purpose of informing a larger RCT.

Most participants in the sample were female and on a secure visa. There were no significant differences between groups for key demographic characteristics or outcome variables at baseline (see Supplementary Table S2). Participants completed a mean of 6.36 modules (range: zero to 11 modules, zero modules: *n* = 11, two to six modules: *n =* 4, six to 10 modules: *n =* 3, 11 modules: *n =* 16).

### Impact of the TYS intervention on self‐stigma and help‐seeking intentions

We report on linear mixed models that investigated the impact of the TYS intervention on SS‐PTSD, SS‐HS, and help‐seeking intentions. Statistics for each linear mixed model are presented in Table 1.

**TABLE 1:** Linear mixed models which include group and time predictors of self‐stigma and help‐seeking outcomes

	Est (*B*)	*SE*	*t*	*df*	*p*
SS‐PTSD					
Intercept	2.33	0.09	25.00	101.16	< .001
Group (TYS)	−0.04	0.13	−0.28	102.60	.784
Group (WLC)					
Time	−0.06	0.02	−3.17	105.68	.002
Group (TYS) x Time	0.06	0.03	2.33	106.92	.022
Group (WLC) x Time					
Effect size	T1: *g* = 0.10, T2: *g* = 0.07, T3: *g* = 0.41				
SS‐HS					
Intercept	2.64	0.13	20.28	115.82	< .001
Group (TYS)	−0.30	0.19	−1.58	115.66	.117
Group (WLC)					
Time	−0.05	0.03	−1.88	107.74	.063
Group (TYS) x Time	0.04	0.04	1.106	108.08	.271
Group (WLC) x Time					
Effect size	T1: *g* = ‐0.17, T2: *g* = ‐0.68, T3: *g* = ‐0.10				
HS intentions					
Intercept	3.59	0.21	17.43	124.89	< .001
Group (TYS)	0.05	.29	0.18	125.13	.860
Group (WLC)					
Time	−0.07	0.05	−1.40	107.52	.165
Group (TYS) x Time	0.04	0.07	−0.55	108.38	.584
Group (WLC) x Time					
Effect size	T1: *g* = 0.10, T2: *g* = 0.13, *g* = T3: 0.38				

*Note*: SS = self‐stigma; PTSD = posttraumatic stress disorder; HS = help‐seeking; *df* = degrees of freedom; TYS = Tell Your Story, WLC = waitlist control.

Linear mixed models revealed that both groups decreased in SS‐PTSD across time, as indicated by the significant time effect. The Group x Time interaction was significant, indicating that the WLC group demonstrated larger decreases in SS‐PTSD across time. Linear mixed models revealed no significant effects for SS‐HS or help‐seeking intentions.

### Impact of the TYS intervention on help‐seeking behavior

At postintervention, the omnibus test revealed that the overall model, which included group as a predictor and help‐seeking behavior as an outcome, was not statistically significant, *G* ^2^(1) = 1.29, *p* = .255. At the follow‐up assessment, the omnibus test was significant, *G* ^2^(1) = 6.72, *p* = .010, indicating an overall statistically significant model. Poisson regression analyses revealed that participants in the TYS group accessed significantly more new sources of help compared to those in the WLC group, *B* = 0.94, *SE* = 0.38, Wald 95% confidence interval (CI) [0.20, 1.68], *p* = .013, *g* = 0.67.

## DISCUSSION

Few interventions have been implemented to reduce self‐stigma and increase help‐seeking among refugees. The present paper reports on the second study to examine TYS, a low‐cost, culturally sensitive, scalable intervention specifically designed for Arabic‐speaking refugees (Nickerson et al., 2020). The findings provide further support for the benefit of the TYS intervention to increase help‐seeking behavior but did not demonstrate support for reducing self‐stigma. Specifically, the findings demonstrate no change in SS‐HS, whereas SS‐PTSD was reduced to a larger extent in the WLC group compared to the TYS group. However, the findings also demonstrate that participants in the TYS group accessed more new sources of mental health support compared to those in the WLC group at 3‐month follow‐up, with a moderate‐to‐large effect. The same pattern of findings was observed when noncompleters were removed from the analysis (see Supplementary Materials). The mixed findings, along with the small sample size, indicate that the results should be interpreted with caution and warrant further investigation in a larger RCT comprising both male and female participants.

The finding that the TYS intervention led to more new sources of accessed help is a replication of the previous RCT and extends the finding, suggesting that this effect is present when women are included in the sample (Nickerson et al., 2020). Further, this effect appeared to last up until 3 months postintervention. This replication and extension provide further support that TYS is a viable approach to increasing help‐seeking behavior, which is especially noteworthy given that behavioral change has been shown to be difficult to achieve with online interventions (Xu et al., 2018). As our measure of help‐seeking indexed new sources of help accessed, the findings suggest that the intervention increased participants’ network of both formal and informal help‐seeking sources. This is especially important given that help‐seeking rates in relation to formal sources are low among refugees and that social ties are often reduced during resettlement in the host country (Aarethun et al., 2021; Satinsky et al., 2019). It is notable that participants who received the TYS intervention showed increases in help‐seeking behavior at the 3‐month follow‐up (i.e., they had accessed more new sources of support over the past 3 months), yet there was not a commensurate change in help‐seeking intentions. This is similar to findings from the previous evaluation of TYS, which also demonstrated an increase in help‐seeking behaviors in the TYS group along with an increase in help‐seeking intentions in the WLC group. This finding may be reflective of the growing body of literature showing a disconnect between intentions and behavior, which can be due to various moderators (Conner & Norman, 2022; Hammer et al., 2019). Perceived behavioral control, in particular, has been found to strongly influence behavior irrespective of the level of intention (Ajzen, 1991). Therefore, it is possible that the change in help‐seeking behavior observed in the TYS group was driven by an increase in perceived behavioral control rather than intentions. This may explain why help‐seeking behavior increased without any change in intentions.

Unexpectedly, though both groups showed decreases in PTSD related self‐stigma (i.e., SS‐PTSD) across time, participants in the WLC group demonstrated larger decreases in SS‐PTSD, whereas there were no effects observed for help‐seeking–related self‐stigma (i.e., SS‐HS). It is possible that participants in the TYS group were better able to identify and appraise their beliefs as self‐stigma related to PTSD because the intervention frequently explores these types of beliefs. Participating in a mental health–related intervention may also have impacted participants’ level of self‐stigma regarding their symptoms. The differential findings between SS‐PTSD and SS‐HS also support research that these constructs are related yet distinct (Tucker et al., 2013).

In sum, the findings suggest that although the TYS intervention was designed to reduce self‐stigma, it was not effective in doing so in this study relative to a waitlist control condition. Instead, the intervention appeared to be beneficial for increasing participants’ networks of help‐seeking sources without commensurate decreases in self‐stigma. A similar finding was observed in a study that aimed to decrease self‐stigma and increase help‐seeking in a sample of young people, which demonstrated that help‐seeking intentions increased, yet there was no difference in self‐stigma between the intervention groups and control group (Howard et al., 2018). Additionally, the findings align with intervention approaches designed to increase help‐seeking without necessarily targeting self‐stigma (Xu et al., 2018). Therefore, in light of the broader literature, the increase in help‐seeking behavior without commensurate decreases in self‐stigma may suggest that TYS has different mechanisms of action to increase help‐seeking behavior. For example, consistent with other effective help‐seeking interventions, TYS may have promoted mental health literacy by facilitating awareness of perceived needs and increasing knowledge about the type of help‐seeking professionals available via psychoeducation.

The findings should be interpreted in the context of the study's limitations. These limitations can inform what can be adapted for future studies to ensure findings are more robust. The main limitation was the relatively small sample size, which reduced power and, therefore, precluded comparisons between men and women; precluded analyses comparing different categories of help‐seeking; and was a smaller sample than the original RCT, which we aimed to replicate and extend. Besides the fact that refugees are a challenging population to reach, the small sample size speaks to a broader issue of recruitment for stigma reduction interventions, wherein it becomes difficult to advertise an intervention for a problem (stigma) that arises because of something that is stigmatized (mental health). Therefore, agreeing to participate can be considered a form of help‐seeking, as the level of stigma is not enough to deter participation (Lindheimer et al., 2020). As such, it is probable that individuals who are experiencing pronounced stigma, which hinders their engagement in mental health research, are not captured in this sort of research. In light of this, future studies should examine the ecological validity of the TYS intervention, perhaps by implementing it within a service such as part of a resettlement package or stepped‐care approach in which it is delivered first, before triaging into more intensive treatments as needed (El‐Haj‐Mohamad et al., 2023). This approach would allow for a more naturalistic sample, and potentially larger sample sizes, to enable gender comparisons and the identification of other moderators that may influence the intervention's effectiveness.

Additionally, the finding that one third of participants did not even start the intervention raises the broader issue of adherence and acceptability. This relatively high rate of nonadherence may be due to self‐stigma affecting participants’ willingness to engage with the intervention, as they may feel ashamed of participating in a mental health–related intervention (Fung et al., 2010; Livingston & Boyd, 2010). Indeed, we found that higher levels of SS‐HS at baseline were associated with fewer modules completed as well as with not starting the intervention (i.e., noncompletion), which suggests that self‐stigma may affect the extent to which individuals engage with the intervention (see Supplementary Table S4). Taken together, this indicates a need for future studies to make increased efforts from the outset to address stigma‐related concerns in the context of completing the intervention.

It is important to note that we also saw a relatively low adherence within this study for participants who engaged with the intervention, with participants completing an average of 6.36 out of 11 modules (i.e., 57.8% of the intervention). Though this reflects an improvement relative to the original TYS study, which saw the completion of an average of 4.76 modules, and is relatively higher than adherence rates (37.5%) reported in a systematic review investigating the efficacy of smartphone interventions, it still suggests further amendments to the intervention are needed to increase engagement (El‐Haj‐Mohamad et al., 2023; Nickerson et al., 2020). For example, participants may have benefited from increased therapist contact, with phone calls instead of email reminders if modules were not completed. Though we elected to have minimal therapist contact in our program to increase scalability, increased therapist contact may have served the purpose of addressing participants’ stigma‐related concerns and resolving any technological difficulties to reduce barriers to participation. It is also possible that 11 modules are too burdensome for participants. Our findings on program usability (Supplementary Table S5) suggest that the social contact component received the highest proportion of participants who rendered a rating of “quite a bit” or “extremely” useful. Therefore, a subsequent investigation may use a dismantling approach to investigate whether particular TYS strategies are more effective at yielding change in help‐seeking. This is particularly important to consider given that the whole intervention facilitated less decline in SS‐PTSD compared to the waitlist control condition. For example, a future study could compare the social contact components to the full TYS intervention to investigate whether social contact alone is equally as effective as the complete intervention in increasing help‐seeking. If so, TYS can be distilled to the most effective components with the potential of increasing engagement, because it would be less burdensome to complete, without stunting the natural decline of SS‐PTSD over time. Despite these issues with adherence and recruitment, the current study found that most participants were satisfied with the program and found it easy to use and understand. This is similar to the reported experience of the male participants in the previous RCT (Nickerson et al., 2020). Finally, the intervention was limited to a focus on PTSS. It would be worthwhile for a future study to adapt the TYS intervention to psychopathology more broadly especially given that cumulative trauma and forced displacement produce a range of mental health difficulties beyond PTSD (Fazel et al., 2005).

In conclusion, this study provides further support for the TYS intervention in increasing help‐seeking behavior, but not decreasing self‐stigma, over a longer‐term follow‐up period and when women are included in the sample. Although the findings warrant further investigation in a larger RCT, this study shows that the TYS intervention has promise for reducing the mental health burden among refugees by enhancing help‐seeking in a population with high mental health rates.

## AUTHOR NOTE

Angela Nickerson was supported by an Australian Health and Medical Research Council Leadership Fellowship.

The authors like to thank all participants for their contributions to this study, particularly the men and women who volunteered to share their stories via the videos—Imad, Mustafa, Olivia, and Yasameen. The authors would also like to thank the Black Dog Institute for their support in hosting the intervention, as well as Armin Awan for filming and producing the video content.

## OPEN PRACTICES STATEMENT

This trial was prospectively registered on the Australia and New Zealand Clinical Trial Registry (Trial ID ACTRN12621001731886). The data set generated and analyzed in the present study is available from the corresponding author upon reasonable request at a.nickerson@unsw.edu.au.

## Supporting information



Supplementary Materials
