# Supplementary material for: An online intervention designed to reduce self‐stigma and increase help‐seeking in Arabic‐speaking refugees with posttraumatic stress symptoms: A randomized controlled trial
Source: J Trauma Stress. 2025 May 30;38(5):832–43. doi: 10.1002/jts.23168 (PMC12551623; doi:10.1002/jts.23168)
Supplement: Supplementary file 1 [file JTS-38-832-s001.odt]

Supplementary Materials

The TYS intervention 

The intervention implemented evidence-based, stigma reduction strategies: social contact, psychoeducation and cognitive re-appraisal of PTSD symptoms and help-seeking concerns. Social contact was facilitated by short videos of two, Arabic-speaking refugees who shared their personal experience of PTSD, stigma and help-seeking using culturally specific expressions. The videos were viewed alongside other interactive activities which incorporated psychoeducation and cognitive reappraisal. In order to adapt the intervention to be suitable for women, the existing intervention was duplicated and male-specific components were adapted. Firstly, new videos were created featuring two, Arabic-speaking refugee women with lived experience. To ensure a level of consistency with the male version, similar themes were discussed such as the prevalence of stigma in the community and the benefit of help-seeking. Additionally, to adapt male specific beliefs included in the intervention (e.g. “it is not masculine to feel stressed”), psychologists experienced in working with Arabic-speaking refugees, as well as female, Arabic-speaking community members who were part of the research term, were consulted to identify more appropriate female specific themes (e.g. “it is selfish to feel stressed”). 

Table S1. TYS module content (adapted from Nickerson et al., 2019).

| Module | Strategies | Content |
| --- | --- | --- |
| 1 Meeting X and Y | Social contact Psychoeducation | Videos introducing both refugees who are followed throughout the intervention Education about mental health difficulties arising from common refugee experiences. |
| 2 Surviving Stress | Psychoeducation | Participants learn about the impact of traumatic experiences and stress on everyday life. Explanation of PTSD symptoms. |
| 3 How does stress affect me? | Social contact Psychoeducation | Participants learn about the symptoms experienced by refugees featured in the videos. Participants identify their own psychological symptoms related to PTSD. |
| 4 Growing stronger | Cognitive reappraisal | Participants identify and reappraise their own negative beliefs about symptoms. |
| 5 Keeping it all inside | Social contact Psychoeducation | Participants observe the refugees in the videos expressing their help-seeking concerns and subsequently identify their own help-seeking concerns. |
| 6 I’d like to talk but… | Cognitive reappraisal | Participants reappraise their own help-seeking concerns. |
| 7 How can talking help | Psychoeducation Social contact | Refugees in the videos share the benefits they have experienced following help-seeking. Participants identify the potential benefits of talking about their problems. |
| 8 Who could I talk to | Psychoeducation Help-seeking planning | Participants identify suitable people they can share their experiences with and seek help from. Participants learn about different professional roles and how each professional may be able to help them. |
| 9 Advice for X | Cognitive reappraisal | Participants are presented with a ‘case study’ of a refugee with a similar background who is struggling with psychological problems. Participants complete an activity in which they help this person challenge their negative beliefs/stigma about mental health problems. |
| 10 Planning action | Cognitive reappraisal Help-seeking planning | Provides an overview of the participant’s own stigma and help-seeking concerns (drawn from endorsed responses to previous activities). Participants complete an activity in which they identify the challenges and benefits to help-seeking. Participants develop a personalized plan of action for help-seeking. |
| 11 Summary | Help-seeking planning Social contact | Participants review their plan of action. Participants watch a summary video of refugees in the video case studies to increase motivation moving forward. |

Table S2. Participant demographics and baseline differences 

| Demographics | Overall sample N = 67 | TYS group n = 33 | WLC group n = 34 | Statistic |
| --- | --- | --- | --- | --- |
| Age | M =44.87 SD=11.62 | M =42.12 SD =10.86 | M =47.53 SD =10.87 | t(65) = 1.95, p = .056 |
| Gender |  |  |  |  |
| Female | 42 (62.7%) | 20 (60.6%) | 22 (64.7%) | χ2  (1) = 0.12 p =.729 |
| Male | 24 (37.3%) | 13 (39.4%) | 12 (35.3%) |  |
| Time in Australia (Years) | M =5.12 SD =2.88 | M =4.51 SD =2.61 | M =5.71 SD =3.03 | t(63) = 1.71, p = .093 |
| Visa status |  |  |  |  |
| Secure (i.e., Australian citizenship, Permanent visa) | 64 (95.5%) | 31 (93.9%) | 33 (97.1%) | χ2  (1) = 0.38 p =.537 |
| Insecure (i.e., Temporary visa, Bridging visa, expired visa or no visa) | 3 (4.5%) | 2 (6.1%) | 1 (2.9%) |  |
| Trauma Exposure (PTEs experienced) | M =5.98 SD =5.00 | M =5.57 SD =4.26 | M =6.34 SD =5.61 | t(56.92) = 0.59 p = .555 |
| PTSD symptoms | M =1.32 SD =0.74 | M =1.36 SD =0.69 | M =1.29 SD =0.79 | t(62) = -0.37 p = .713 |
| Self-stigma of PTSD | M =2.29 SD =0.49 | M =2.32 SD =0.53 | M =2.27 SD =0.45 | t(62) = -0.40 p = .688 |
| Self-stigma of seeking help | M =2.47 SD =0.71 | M =2.41 SD =0.78 | M =2.53 SD =0.61 | t(63) = 0.68 p = .497 |
| Help-seeking intentions | M =3.62 SD =1.21 | M =3.68 SD =1.24 | M =3.55 SD =1.21 | t(64) = -0.43 p = .670 |
| Help-seeking behaviour (number of help-seeking sources accessed in the past 2 weeks prior to baseline) | M =0.82 SD =0.90 | M =0.78 SD =0.94 | M =0.88 SD =0.87 | t(62) = 0.41 p = .681 |

Note. PTS symptoms were measured using the PDS, self-stigma of PTSD was measured by the ISMI (adapted for PTSD), self-stigma of seeking help was measured using the SSOSH scale and help-seeking intentions was measured using the GHSQ. Further details are included in the ‘Measures’ section. 

Analyses which exclude non-completers 

The impact of the TYS intervention on self-stigma and help-seeking intentions. We report on linear mixed models which investigated the impact of the TYS intervention on SSPTSD, SSOSH and help-seeking intentions while excluding non-completers (TYS: n=22 WLC: n =34). Statistics of each linear mixed model are presented in Table C. 

| Table S3. Linear Mixed models which include group and time as a predictor for self-stigma and help-seeking outcomes excluding non-completers. |  |
| --- | --- |
|  | Est (B) | SE | df | t | p |  |
| SSPTSD |  |  |  |  |  |  |
| Intercept | 2.34 | .09 | 83.46 | 25.06 | <.001 |  |
| Group (TYS) | -0.11 | .15 | 85.03 | -0.71 | .480 |  |
| Group (WLC) | - | - |  |  |  |  |
| Time | -0.06 | .02 | 90.54 | -3.24 | .002 |  |
| Group (TYS) x time | 0.07 | .03 | 91.29 | 2.50 | .014 |  |
| Group (WLC) x time | - | - |  |  |  |  |
| Effect size (g) | T1: -0.11  T2: 0.13  T3: 0.44 |
|  |  |  |  |  |  |  |
|  |  |  |  |  |  |  |
| SSOSH |  |  |  |  |  |  |
| Intercept | 2.64 | .12 | 100.06 | 22.41 | <.001 |  |
| Group (TYS) | -0.42 | .19 | 99.30 | -2.22 | .029 |  |
| Group (WLC) | - | - |  |  |  |  |
| Time | -0.05 | .03 | 93.66 | -2.04 | .044 |  |
| Group (TYS) x time | 0.04 | .04 | 93.42 | 1.00 | .337 |  |
| Group (WLC) x time | - | - |  |  |  |  |
| Effect size (g) | T1: -051  T2: -0.58  T3: -0.30 |
|  |  |  |  |  |  |  |
|  |  |  |  |  |  |  |
| Help-seeking intentions |  |  |  |  |  |
| Intercept | 3.59 | .21 | 105.19 | 17.19 | <.001 |  |
| Group (TYS) | -0.07 | .33 | 105.27 | -0.22 | .826 |  |
| Group (WLC) | - | - |  |  |  |  |
| Time | -0.07 | .05 | 90.52 | -1.37 | .173 |  |
| Group (TYS) x time | 0.03 | .08 | 91.12 | 0.32 | .751 |  |
| Group (WLC) x time | - | - |  |  |  |  |
| Effect size (g) | T1: -0.01  T2: -0.04  T3: 0.21 |

SSPTSD. Linear mixed models revealed that both groups decreased in SSPTSD across time (as indicated by the significant time effect). The group x time interaction was significant, indicating that the waitlist group demonstrated greater decreases in SSPTSD across time.  

SSOSH. Linear mixed models revealed that both groups decreased in SSOSH across time (as indicated by the significant time effect). There was a significant group effect whereby the TYS group displayed lower levels of SSOSH overall. The group x time interaction was not significant. 

Help-seeking intentions. Linear mixed models revealed no significant effects for help-seeking intentions.  

The impact of the TYS intervention on help-seeking behaviour. 

At post-assessment, the omnibus test revealed that the overall model which included group as a predictor and actual help-seeking behaviour as an outcome, was not statistically significant (G2 =0.66 df=1 p=.417). At the follow-up assessment, the omnibus test was significant (G2=4.58 df =1 p=.032) indicating an overall statistically significant model. Poisson regression analyses revealed that the TYS group accessed significantly more, new sources of help compared to the WLC group (B=0.86, SE=.41, 95% Wald CI 0.06 to 1.66 p=.035, g=0.63).

Non-completers versus participants who completed at least one module

	To assess for any differences in those who engaged in the intervention versus those who did not, non-completers and participants who engaged with at least one module were compared on baseline demographics and clinical outcome measures using t tests and chi-square tests. As can be seen in Table D, independent samples t tests revealed that, compared to participants who completed at least one module, non-completers reported significantly higher SSOSH and more years in Australia. There were no significant differences on age, gender, PTS symptoms and help-seeking intentions. 

Table S4. Comparisons of non-completers versus participants who completed at least one module

| Variable | Non-completers n=11 | Participants who completed at least one module n=22 | Statistic |
| --- | --- | --- | --- |
| Age | M=40.18 SD=10.53 | M=43.09 SD=11.13 | t(31)= -0.72, p= .477 |
| Gender |  |  |  |
| Female | 6 (54.5%) | 14 (63.6%) | χ2  (1)= 0.25 p=.614 |
| Male | 5 (45.5%) | 8 (36.4%) |  |
| Time in Australia (Years) | M=5.95 SD=1.87 | M=3.76 SD=2.66 | t(27.16)= 2.69, p=.012 |
| PTS symptoms | M=1.46 SD=0.69 | M=1.32 SD=0.70 | t(29)= 0.51, p=.615 |
| Self-stigma of PTSD | M=2.52 SD=0.50 | M=2.22 SD=0.52 | t(30)=1.56, p=.130 |
| Self-stigma of seeking help | M=2.82 SD=0.88 | M=2.20 SD=0.66 | t(31)= 2.26, p=.031 |
| Help-seeking intentions | M=3.70 SD=1.29 | M=3.87 SD=1.85 | t(31)= -0.27, p=.791 |

Note. PTS symptoms were measured using the PDS, self-stigma of PTSD was measured by the ISMI (adapted for PTSD), self-stigma of seeking help was measured using the SSOSH scale and help-seeking intentions was measured using the GHSQ. Further details are included in the ‘Measures’ section.

Self-stigma and number of modules completed 

We were also interested in investigating whether self-stigma impacted engagement with the intervention. To do so, Pearson’s correlations investigated whether baseline SSPTSD and SSOSH were correlated with number of modules completed. Pearson’s correlations revealed that the mean score of SSOSH at baseline was negatively correlated with number of modules completed (r= -0.35, p=0.044) suggesting that greater SSOSH was associated with fewer modules completed. The correlation between SSPTSD and number of modules completed was not significant.

Program Usability

Program usability was assessed using 13 items measuring the following domains: mode of access and the extent to which the website was easy to understand; provided useful information and advice; interesting and engaging; relevance of information; intervention satisfaction and enjoyment. The usefulness of each technique was also assessed (videos, information and interactive activities). Finally, the measure assessed if the participant felt the website took too long to do and whether they would recommend it to a friend. Each item was rated on a continuous scale which differed depending on the item. The proportion of participants who endorsed different response options are reported for each item individually.

For all data on program usability, see Table E. Of the 33 participants in the intervention condition, 16 completed the survey on program usability. The majority of participants ‘agreed’ or ‘strongly agreed’ that they were satisfied with the program (81.3%) and found it either ‘easy’ or ‘very easy’ to use (62.5%) and ‘agreed’ or ‘strongly agreed’ that it was easy to understand (87.6%). This aligns with the program usability in the original RCT with a sample of men whereby 100% of participants indicated the program was ‘easy’ or ‘very easy’ to use and 88.7% of participants ‘agreed’ or ‘strongly agreed’ that the program was easy to understand (Nickerson, et al., 2019). The majority of participants endorsed that they enjoyed the website ‘quite a bit’ or ‘extremely’ (68.8%) and approximately three quarters of participants were likely to recommend the website to a friend (75.1%). This also compares to the previous RCT whereby 56.8% of participants enjoyed the program ‘quite a bit’ or ‘extremely’ (Nickerson et al., 2019).

Table S5. Program Usability data completed by 16 participants who were in the TYS condition

| Item | Scale | Mean (SD) | Proportion |
| --- | --- | --- | --- |
| How did you usually access the Tell Your Story website? | N/A | N/A | Computer/laptop: 12.5% Smartphone: 87.5% |
| How easy was the website to use on your device? | 1 = Very easy 5 = Very difficult | 2.19 (1.17) | Easy, very easy n = 10, 62.5% |
| Overall, I was satisfied with the Tell Your Story Website | 1 = Strongly disagree 5 = Strongly Agree | 4.25 (0.93) | Agree, Strongly Agree n = 13, 81.3% |
| The website was easy to understand | 1 = Strongly disagree 5 = Strongly Agree | 4.38 (0.89) | Agree, Strongly Agree n = 14, 87.6% |
| The website provided useful information and advice | 1 = Strongly disagree 5 = Strongly Agree | 4.19 (0.83) | Agree, Strongly Agree n = 14, 87.5% |
| I felt that the information provided was relevant to me | 1 = Strongly disagree 5 = Strongly Agree | 4.00 (1.13) | Agree, Strongly Agree n = 10, 66.7% |
| The website took too long to do | 1 = Strongly disagree 5 = Strongly Agree | 2.69 (0.95) | Agree* n = 4, 25% |
| Overall, how interesting and engaging was the website? | 1 = Not at all 4 = Extremely | 2.69 (1.01) | Quite a bit, Extremely n = 9, 56.3% |
| How much did you enjoy using the website? | 1 = Not at all 4 = Extremely | 3.00 (0.97) | Quite a bit, Extremely n = 11, 68.8% |
| How useful did you find the videos of men or women talking about their personal experiences? | 1 = Not at all 4 = Extremely | 2.94 (1.00) | Quite a bit, Extremely n = 10, 62.5% |
| How useful did you find the information (for example, information about the impact of stress, the benefits of talking to someone and the different people you could talk to)? | 1 = Not at all 4 = Extremely | 2.81 (0.98) | Quite a bit, Extremely n = 9, 56.3% |
| How useful did you find the interactive activities and tasks (for example, considering your beliefs about stress and talking to someone, making a plan about who you might talk to and how)? | 1 = Not at all 4 = Extremely | 2.67 (0.98) | Quite a bit, Extremely n = 7, 46.7% |
| Would you recommend the website to a friend? | 1 = Extremely unlikely  7 = Extremely likely | 4.69 (1.78) | Likely, Extremely Likely n = 12, 75.1% |

Note. *Participants did not endorse ‘strongly agree’ for this item.
